# An exploratory study for tuft cells in the breast and their relevance in triple-negative breast cancer: the possible relationship of SOX9

**DOI:** 10.1186/s12885-023-10949-5

**Published:** 2023-05-13

**Authors:** Yosuke Yamada, Ronald Simon, Kosuke Iwane, Yuki Nakanishi, Yasuhide Takeuchi, Akihiko Yoshizawa, Masahiro Takada, Masakazu Toi, Hironori Haga, Alexander Marx, Guido Sauter

**Affiliations:** 1 Department of Diagnostic Pathology, Kyoto University Hospital, 54 Shogoin Kawahara-Cho, Sakyo-Ku, Kyoto, 606–8507 Japan; 2 Institute of Pathology, University Medical Center Hamburg-Eppendorf, Hamburg, Germany; 3 Department of Gastroenterology and Hepatology, Kyoto University Graduate School of Medicine, Kyoto, Japan; 4 Department of Breast Surgery, Kyoto University Graduate School of Medicine, Kyoto, Japan; 5 Institute of Pathology, Mannheim and Medical Faculty Mannheim, University Medical Centre, Heidelberg University, Mannheim, Germany

**Keywords:** Breast neoplasms, POU2F3, SOX9, Triple-negative breast neoplasms, Tuft cells

## Abstract

**Background:**

Breast cancer is highly heterogeneous, suggesting that small but relevant subsets have been under-recognized. Rare and mainly triple-negative breast cancers (TNBCs) were recently found to exhibit tuft cell-like expression profiles, including POU2F3, the tuft cell master regulator. In addition, immunohistochemistry (IHC) has identified POU2F3-positive cells in the normal human breast, suggesting the presence of tuft cells in this organ.

**Methods:**

Here, we (i) reviewed previously identified POU2F3-positive invasive breast cancers (*n* = 4) for POU2F3 expression in intraductal cancer components, (ii) investigated a new cohort of invasive breast cancers (*n* = 1853) by POU2F3-IHC, (iii) explored POU2F3-expressing cells in non-neoplastic breast tissues obtained from women with or without *BRCA1* mutations (*n* = 15), and (iv) reanalyzed publicly available single-cell RNA sequencing (scRNA-seq) data from normal breast cells.

**Results:**

Two TNBCs of the four previously reported invasive POU2F3-positive breast cancers contained POU2F3-positive ductal carcinoma in situ (DCIS). In the new cohort of invasive breast cancers, IHC revealed four POU2F3-positive cases, two of which were triple-negative, one luminal-type, and one triple-positive. In addition, another new POU2F3-positive tumor with a triple-negative phenotype was found in daily practice. All non-neoplastic breast tissues contained POU2F3-positive cells, irrespective of *BRCA1* status. The scRNA-seq reanalysis confirmed POU2F3-expressing epithelial cells (3.3% of all epithelial cells) and the 17% that co-expressed the other two tuft cell-related markers (SOX9/AVIL or SOX9/GFI1B), which suggested they were bona fide tuft cells. Of note, SOX9 is also known as the “master regulator” of TNBCs.

**Conclusions:**

POU2F3 expression defines small subsets in various breast cancer subtypes, which can be accompanied by DCIS. The mechanistic relationship between POU2F3 and SOX9 in the breast warrants further analysis to enhance our understanding of normal breast physiology and to clarify the significance of the tuft cell-like phenotype for TNBCs.

**Supplementary Information:**

The online version contains supplementary material available at 10.1186/s12885-023-10949-5.

## Background

Due to substantial heterogeneity in breast cancer, subclassification is necessary for appropriate treatment, which can be made by immunohistochemistry (IHC) with formalin-fixed paraffin-embedded tissues [1]. Of the molecular subtypes of breast cancer, the triple-negative subtype (triple-negative breast cancers [TNBCs]), in which hormone receptors (HRs: estrogen receptor [ER] and progesterone receptor [PR]) and HER2 are not expressed, is reported to be the most diverse group [2, 3]. This cancer type can thus be subclassified further for advancing personalized medicine. Tuft cells are chemosensory cells that occur at tissue-environmental interfaces, such as in the epithelial lining of the intestine [4–6], but were also recently detected in non-mucosal sites such as the thymus [7, 8]. Huang et al. first discovered cancers with tuft cell-like signatures (i.e., the expression of tuft cell markers, e.g., the master regulator POU2F3 [9], and GFI1B, TRPM5, SOX9, CHAT, and AVIL [10]) as a small subset of small cell lung cancer (SCLC).

Subsequently, tuft cell-like cancers were also reported in major non-small cell lung cancer (NSCLC) histotypes and thymic carcinomas [11, 12]. Furthermore, this type of cancer was found to rarely occur in extra-thoracic organs as biologically distinct subsets [13, 14], which often exhibit poorly differentiated histology and overexpress the well-known oncogenes *BCL2* [13, 14] and *KIT* [13]. In addition, tuft cell-like cancers exhibit unique expression profiles depending on the primary sites and often display the triple-negative subtype in the breast [13]. A tuft cell-like phenotype might be therapeutically relevant because tuft cell-like SCLC cell lines have been reported to be highly sensitive to PARP inhibitors [15–17], and extra-thoracic tuft cell-like cancers often express SLFN11 [13], a promising biomarker of PARP inhibitor susceptibility [17, 18].

Interestingly, tuft cell-like cancers can occur in organs where the presence of tuft cells has not been reported. In this context, a small number of POU2F3-positive cells was detected in the breast [13]. Based on this unique finding, we assumed that a possible link between tuft cell-like cancers and normal tuft cells in the breast would merit further investigation for dissecting breast cancers, particularly TNBCs, in relation to normal cellular physiology. Hopefully, this may ultimately advance personalized breast cancer therapies.

## Methods

### Analysis of breast cancers and normal breast tissues

In this study, we reviewed four invasive breast carcinomas with POU2F3 expression that we had previously reported [13]. However, to investigate possible in situ components that were not included in previous tissue microarrays (TMAs), we used tumor full-face sections. In addition, we analyzed a new cohort of 1853 invasive breast cancers (Table S1) embedded in TMAs that were established by the Institute of Pathology, University Medical Center Hamburg-Eppendorf, Hamburg, Germany. The TMAs consisted of 2144 cores/cases; 1853 of the 2144 cores (86%) could be evaluated for POU2F3 immunostaining and were enrolled in this study.

Patient age, gender, survival data, histological subtypes, the expression status of ER, PR, and HER2, and the Ki-67 labeling index in the POU2F3-positive cases were retrieved from the pathological archives. In addition, we described the clinicopathological features of one POU2F3-positive breast cancer, which was newly found in daily practice. We also examined 15 non-neoplastic and five fibroadenoma tissues. The non-neoplastic tissues were marginal tissues of surgically resected specimens for breast cancers or prophylactic mastectomy samples; five specimens were from patients in their 30 s (premenopausal), five in their 60 s (postmenopausal), and five were from patients with *BRCA1* mutations.

### Immunohistochemistry

We examined the protein expression status by IHC using automated immunostainers (Benchmark Ultra, Roche Diagnostics, Rotkreuz, Switzerland, with ultraView, for BCL2, CK5, ER, KIT, POU2F3(s); BOND-III, Leica Biosystems, Nussloch, Germany, with Bond Polymer Refine Detection kit, for SOX9). The primary antibodies were directed to BCL2 (S66, ready to use, Roche Diagnostics), CK5 (SP27, ready to use, Roche Diagnostics), ER (SP1, ready to use, Roche Diagnostics), KIT (polyclonal, dilution 1:200, Agilent Technologies, Santa Clara, CA, USA), POU2F3 (polyclonal, dilution 1:100, Sigma-Aldrich, St. Louis, MO, USA), POU2F3 (E5N2D, dilution 1:100, Cell Signaling Technology, Danvers, MA, USA), and SOX9 (polyclonal, dilution 1:1000, Merck, Darmstadt, Germany). For POU2F3-IHC, the polyclonal anti-POU2F3 antibody was used for breast cancer TMA tissues, and the novel monoclonal anti-POU2F3 antibody, released while conducting this study, was used for non-neoplastic and fibroadenoma tissues, and for one newly identified POU2F3-positive case in routine practice. Human skin was used as a positive control, and POU2F3 expression in cancer cells was considered positive if at least 10% of tumor cells were immunoreactive [12].

### Reanalysis of a publicly available single-cell RNA sequencing dataset

We reanalyzed a recent scRNA-seq dataset of normal human breast tissues from 11 donors that was originally reported to comprise 23 subclusters of breast epithelial cell types (GSE164898) [19]. We read the scRNA-seq data using R4.0.3 (https://www.R-project.org/) and package Seurat (ver. 4.0.2). We excluded the following genes and cells because the obtained data were unreliable; genes that were expressed in < 5 cells; cells that expressed < 200 genes or > 7000 genes; cells with counts of mitochondrial genes occupying > 10% of all the counts. First, we log-normalized and integrated all scRNA-seq data using the FindIntegrationAnchors and IntegrateData functions and performed dimension reduction by PCA and UMAP. Second, we conducted clustering analyses using the FindNeighbors and FindClusters functions. Gene expression was plotted using the FeaturePlot function. Clusters comprised of epithelial cells were identified by the expression of EPCAM and ITGA6 [20]. We reassorted a total of 11,831 epithelial cells into 23 clusters.

We also performed pseudo-bulk analysis to address the gene expression signature of POU2F3-expressing cells. In this analysis, cells in which one or more reads were mapped to the *POU2F3* gene were interpreted as POU2F3-expressing/positive (*n* = 390). Subsequently, the genes that the POU2F3-positive group expressed significantly more frequently than the POU2F3-negative group were extracted. This analysis failed to extract genes that were significantly expressed in the POU2F3-positive group; thus, we alternatively focused on clusters enriched with POU2F3-positive cells. Then, we investigated genes that were significantly expressed in the cells in the clusters.

## Results

### Ductal carcinoma in situ expresses POU2F3

**Fig. 1:**
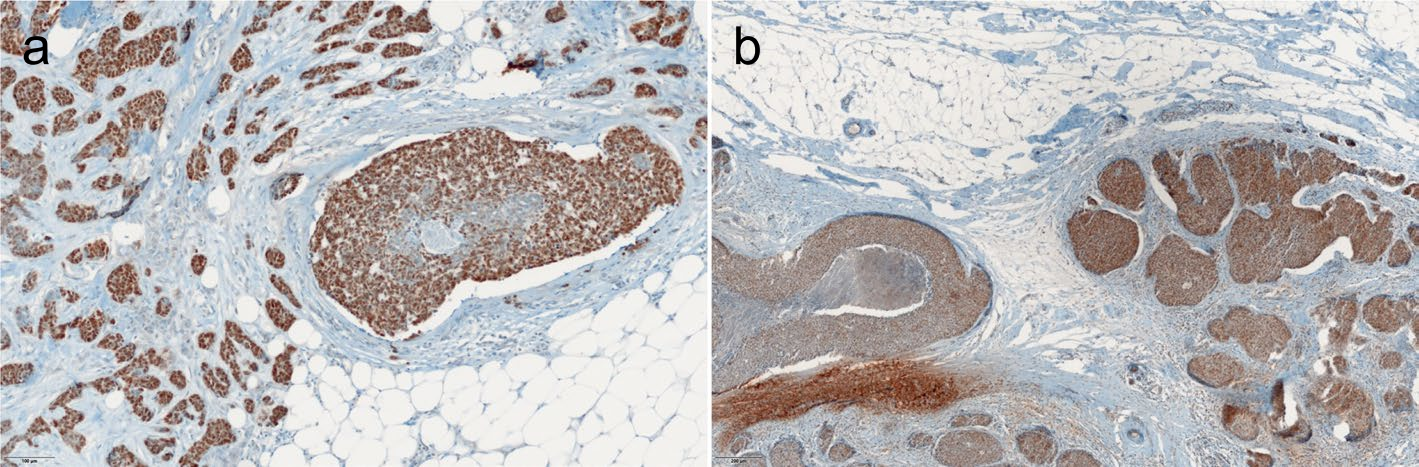
POU2F3 expression of ductal carcinoma in situ (DCIS) components in POU2F3-positive breast cancers (a review of previously reported cases [13]). (**a**) Case 1, (**b**) Case 2. Both cases contain DCIS components. As with invasive lesions, the DCISs are positive for POU2F3 (a, b: immunohistochemistry)

Previous TMA-based analysis identified four POU2F3-positive breast cancers, all of which were “invasive” ductal breast carcinomas [13]. To find out whether POU2F3 was also expressed in the respective “non- or pre-invasive” lesion, i.e., ductal carcinoma in situ (DCIS), we investigated full-face sections of the previously identified four invasive POU2F3-positive breast cancer cases. Two of the four cancers contained both invasive and DCIS components, and the latter also expressed POU2F3. The staining intensity of POU2F3 between in situ and invasive components was comparable for the two cases (Fig. 1a,b). This result suggested that POU2F3 expression can occur in the early stages of cancer development and was possibly related to a special line of differentiation (or histogenesis) but was not associated with invasion.

### POU2F3 expression in a large new cohort of invasive breast carcinomas

**Fig. 2:**
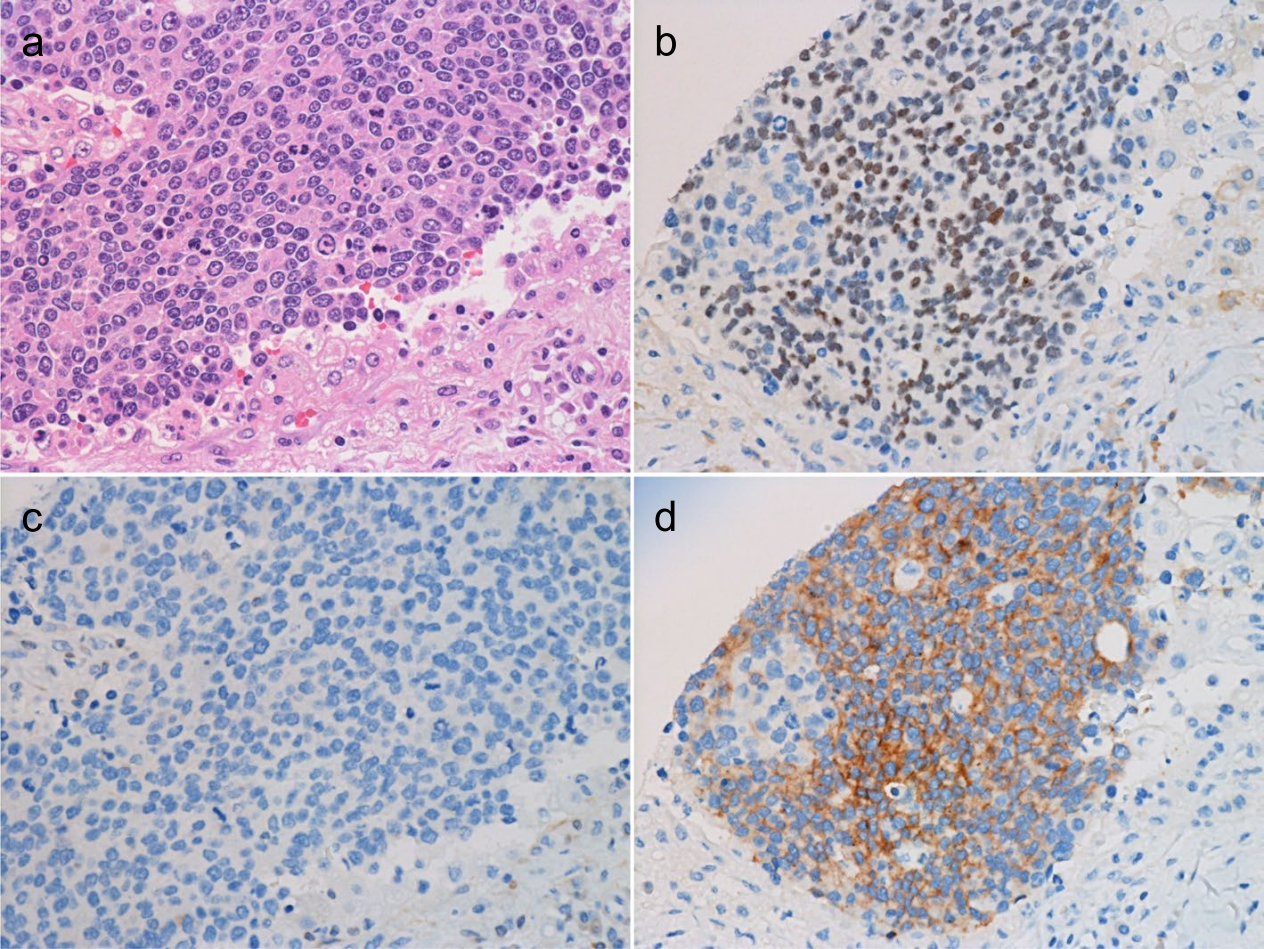
Pathological features of POU2F3-positive invasive breast carcinomas in our new cohort. (**a**–**d**) Tumor no. 1 (Table 1). This tumor shows high-grade histology with rosette-like structures and displays the triple-negative subtype (**a**). The neoplastic cells are positive for POU2F3 (**b**) and KIT (**d**), but negative for BCL2 (**c**) (a: hematoxylin and eosin [H&E] staining; b-d: immunohistochemistry)

**Fig. 3:**
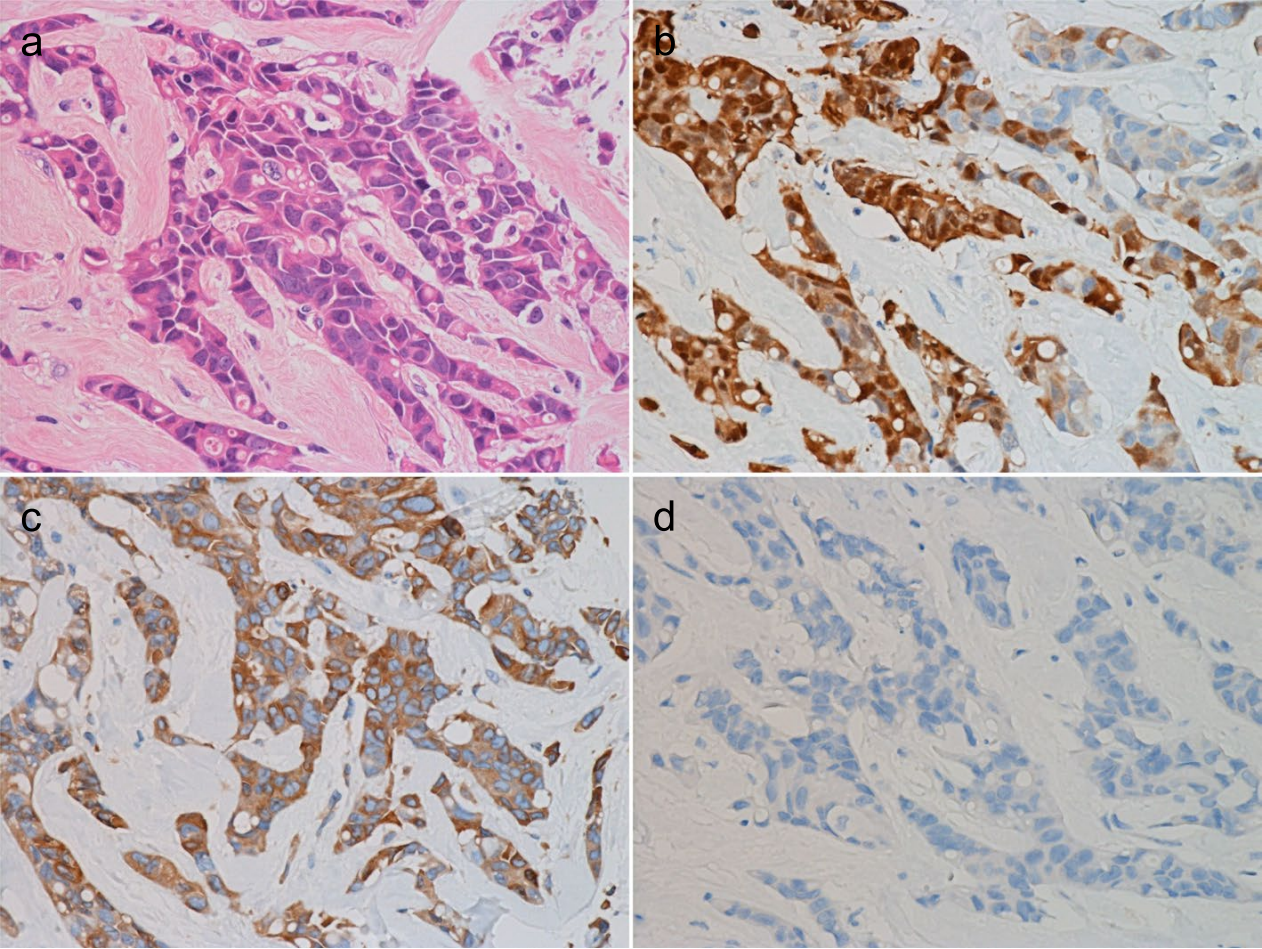
Pathological features of POU2F3-positive invasive breast carcinomas in our new cohort. (**a**–**d**) Tumor no. 4 (Table 1). This tumor is classified as the luminal-HER2 subtype. The neoplastic cells express POU2F3 (**b**) and BCL2 (**c**). KIT is negative for the tumor (**d**) (a: H&E staining; b-d: immunohistochemistry)

We then examined a large, independent breast cancer cohort and found that four of the 1853 cases (0.22%) were positive for POU2F3 based on IHC (Figs. 2 and 3). Two cases were triple-negative (Fig. 2), one was luminal A, and one belonged to the luminal-HER2 (triple-positive) subtype (Fig. 3), the latter being the first such case in which POU2F3 was found to be expressed. This result mirrors our previous finding of increased representation of POU2F3-positive cancers in the triple-negative subtype, a rare POU2F3-positive luminal-type case, and the absence of POU2F3 expression in cases of the HER2-subtype [13]. The staining intensity of POU2F3 was varied and stronger in one of the TNBC cases and in the luminal-HER2 case (Figs. 2 and 3) among the four cases; the strongest expression was observed in one of the TNBC cases reviewed in this study (i.e., with the DCIS component; Fig. 1). Both triple-negative cases showed high-grade histology with high proliferative activities. One of the two tumors formed rosette-like structures (Fig. 2a) but was negative for neuroendocrine markers, i.e., chromogranin A and synaptophysin (data not shown).

BCL2 and KIT, i.e., oncogenes/proteins commonly overexpressed in tuft cell-like carcinomas [13, 14], were expressed in one of the two triple-negative cases (Fig. 2c,d). BCL2 was also expressed in the luminal and luminal-HER2 cases (Fig. 3c,d). Regarding the subtypes of TNBCs, a basal marker CK5 [21] was negative in all the cases (data not shown). In routine practice, we also found one POU2F3-positive invasive breast cancer that exhibited the triple-negative phenotype and CK5 positivity. The clinicopathological features of the five cases are summarized in Table 1. The prognostic impact of POU2F3 positivity could not be assessed due to the small number of POU2F3-positive cases assessed.

**Table 1:** Clinicopathological features of POU2F3-positive invasive breast carcinomas in a new cohort (*n* = 1853 [TMA] + 1 [biopsy])

No	Age	Gender	pT	pN	FU-M	Survival	Grade	Subtypes	POU2F3	BCL2	KIT	ER	PR	HER2	Ki-67	CK5
1	61	F	NA	NA	NA	NA	3	Triple-negative	60	0	80	N	N	N	70	N
2	56	F	NA	NA	37	Alive	3	Triple-negative	10	40	0	N	N	N	40	N
3	66	F	2	0	NA	NA	1	Luminal	10	80	0	P	P	N	5	N
4	72	F	1	0	NA	NA	2	Luminal-HER2	30	80	0	P	P	P	5	N
5	38	F	NA	NA	1	Alive	3	Triple-negative	10	50	10	N	N	N	80	90

No.1–4: TMA samples; No.5: biopsy specimen

*FU-M* Follow-up (month), *Grade* Histological Nottingham grade, *Ki-67 LI* Ki-67 labeling index, *NA* Not available, *N* Negative, *P* Positive

### Non-neoplastic breast tissue contains POU2F3-positive cells

**Fig. 4:**
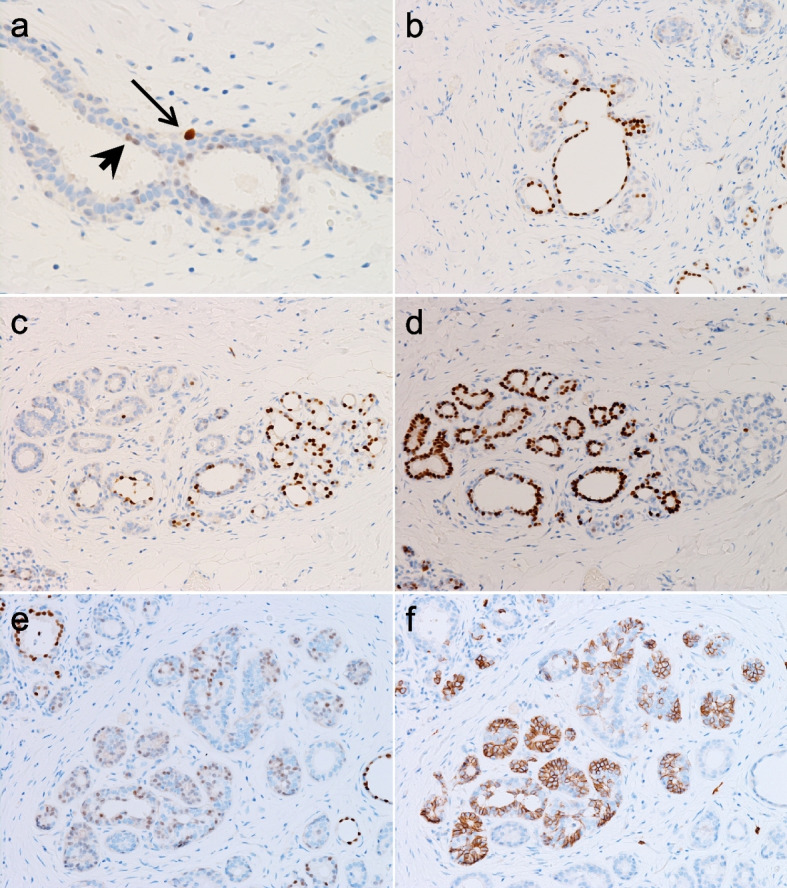
Pathological features of POU2F3-positive cells in the human non-neoplastic breast. POU2F3-positive cells in the non-neoplastic breast can be classified into two types. One is near myoepithelial cells with moderate to strong intensity (**a**, arrow). The other one is close to the lumen with generally weak intensity (**a**, arrowhead) but sometimes shows bright intensity, for example, when they are in ducts/acini with cystic changes (**b**). In some lobules, POU2F3 (**c**) and ER (**d**) are almost mutually expressed. Conversely, some POU2F3-positive cells (**e**) seemingly co-expressed KIT (**f**) (a-f: immunohistochemistry)

Next, we examined POU2F3-positive cells in non-neoplastic breast tissues and confirmed that all the tissues, irrespective of patient age and *BRCA1* status, contained POU2F3-positive cells, mainly in ductal portions (Fig. 4). These POU2F3-positive cells were generally few in number (< 5% in epithelial cells), consistent with our preliminary analysis [13]. However, perhaps because we used a recently released and apparently more sensitive anti-POU2F3 rabbit monoclonal antibody, we found that POU2F3-positive cells could be divided into at least two types. One type was dispersed close to myoepithelial cells and generally showed moderate to strong staining intensity (henceforth, type 1 POU2F3-positive cells), which we had already noted in a previous paper [13] (Fig. 4a). The second type occurred close to the lumen, generally exhibited weak but sometimes strong POU2F3 expression, and could form clusters (henceforth, type 2 POU2F3-positive cells). The staining intensity of type 2 cells seemed stronger when they consisted of ducts/acini showing cystic changes (Fig. 4a,b).

**Fig. 5:**
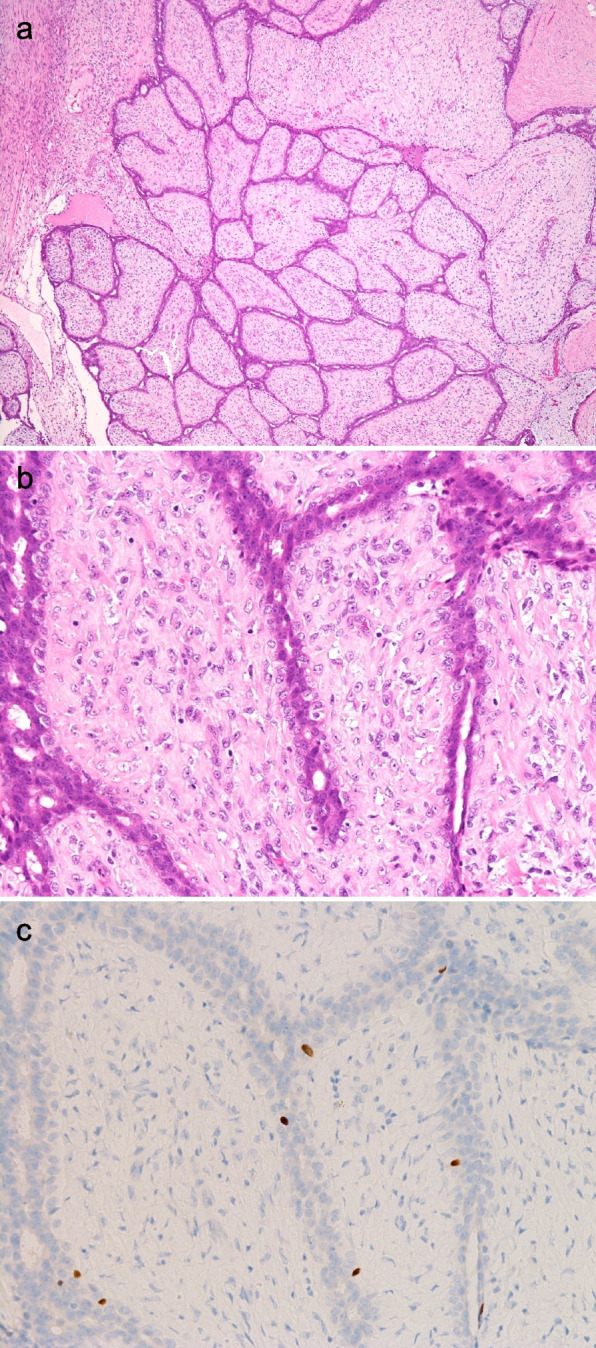
POU2F3 expression in fibroadenoma. Neoplastic epithelial cells focally express POU2F3 (**a**, **b**: H&E staining; c: immunohistochemistry)

We then compared the distribution of POU2F3-positive cells and cells expressing ER, CK5 (a basal marker), and oncogenes often expressed in tuft cell-like cancers (BCL2 and KIT [13]) to ask whether the POU2F3-positive cells had a phenotype similar to tuft cell-like breast cancers. ER was expressed in some (primarily type 2) POU2F3-positive cells, but in some lobules an almost mutually exclusive pattern was observed (Fig. 4c,d). On the other hand, some POU2F3-positive cells seemingly co-expressed CK5 (not shown) and KIT (Fig. 4e,f). BCL2 was expressed in most epithelial cells without correlating with POU2F3 (not shown). All five fibroadenomas contained a small number of POU2F3-positive cells without marked differences among the cases. These positive cells were generally scattered and corresponded mainly to type 1 cells in the non-neoplastic tissues, particularly in the intracanalicular subtype (Fig. 5a-c).

### Reanalysis of single-cell RNA sequencing for non-neoplastic breast epithelial cells detects POU2F3-expressing cells

**Fig. 6:**
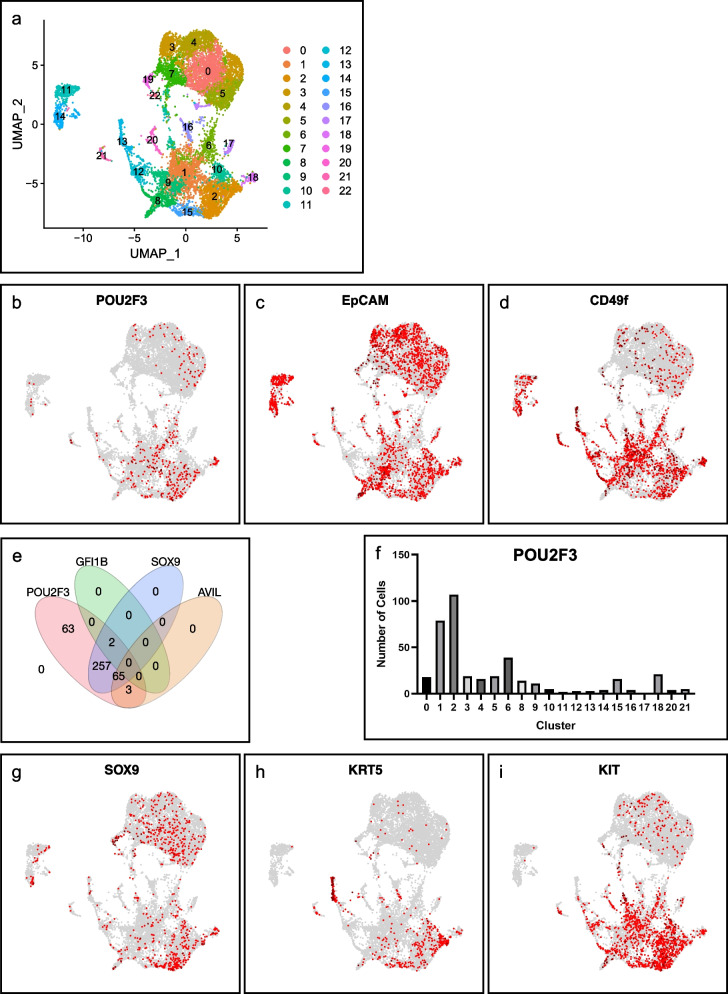
Reanalysis of single-cell RNA sequencing for normal human breast [19]. Epithelial cells in the non-neoplastic breast were separated into 23 subclusters in our analysis (**a**). Although the number was small, POU2F3-expressing cells were detected (**b**; *n* = 390 out of 11,831 cells [3.3%]). These POU2F3-positive cells were either luminal progenitors (CD49f + /EpCAM +) or mature luminal cells (CD49f − /EpCAM +), but almost no basal/stem cells (CD49f + /EpCAM −) [13] (**c**, **d**). Small subsets of POU2F3-expressing cells also co-expressed other tuft cell markers; 65 co-expressed SOX9/AVIL and 2 SOX9/GFI1B (**e**). POU2F3-expressing cells were enriched in clusters 1 and 2; 48% of all the POU2F3-expressing cells (**f**). The distribution of SOX9- (**g**), KRT5- (**h**), and KIT- (**i**) expressing cells. The cells in combined clusters 1 and 2 markedly express these three genes (Table S3)

To validate the immunohistochemical findings in POU2F3-positive cells in the non-neoplastic breast, we reanalyzed publicly available data on single-cell RNA sequencing for non-neoplastic breast epithelial cells in humans [19]. As with the original study [19], breast epithelial cells were separated into 23 clusters (Fig. 6a). The presence of POU2F3-expressing cells was confirmed (390 out of 11,831 cells, 3.3%; Fig. 6b), and these were mainly categorized into luminal progenitors (EPCAM + /ITGA6 +) or mature luminal cells (EPCAM + /ITGA6 −), but seldom into basal/stem cells (EPCAM − /ITGA6 +) [20] (Fig. 6b–d). We then investigated the gene expression signature, particularly the tuft cell-like signature, of POU2F3-expressing cells using pseudo-bulk analysis between POU2F3-positive cells (*n* = 390) and POU2F3-negative cells (*n* = 11,441). However, unfortunately, no genes were extracted using this analysis, perhaps due to the small number of POU2F3-expressing cells and/or heterogeneity within the POU2F3-positive and/or -negative cells, as suggested by the IHC analysis on normal breast tissues.

Thus, we individually searched the POU2F3-expressing cells that also expressed five other representative tuft cell-related genes, i.e., *GFI1B*, *TRPM5*, *SOX9*, *CHAT*, and *AVIL* [10, 22]. Consequently, we found that 67 cells (17%) expressed the other two tuft cell genes, which might be bona fide tuft cells. All cells expressed *SOX9*, while 65 of the 67 cells expressed *AVIL,* and the other two cells expressed *GFI1B* (Fig. 6e and Table S2). Although the pseudo-bulk analysis failed to identify characteristic expression profiles of POU2F3-expressing cells, we found that they were enriched in the two adjacent clusters, clusters 1 and 2 (186 out of 390; 48%; Fig. 6f). Accordingly, we investigated expression profiles of cells within clusters 1 and 2 and found that 275 genes were expressed significantly in these cells compared with cells in other clusters, including *SOX9*, *KRT5*, a basal marker [21], and *KIT*, which is often overexpressed in tuft cell-like cancers [11, 13] (Table S3 and Fig. 6g-i).

**Fig. 7:**
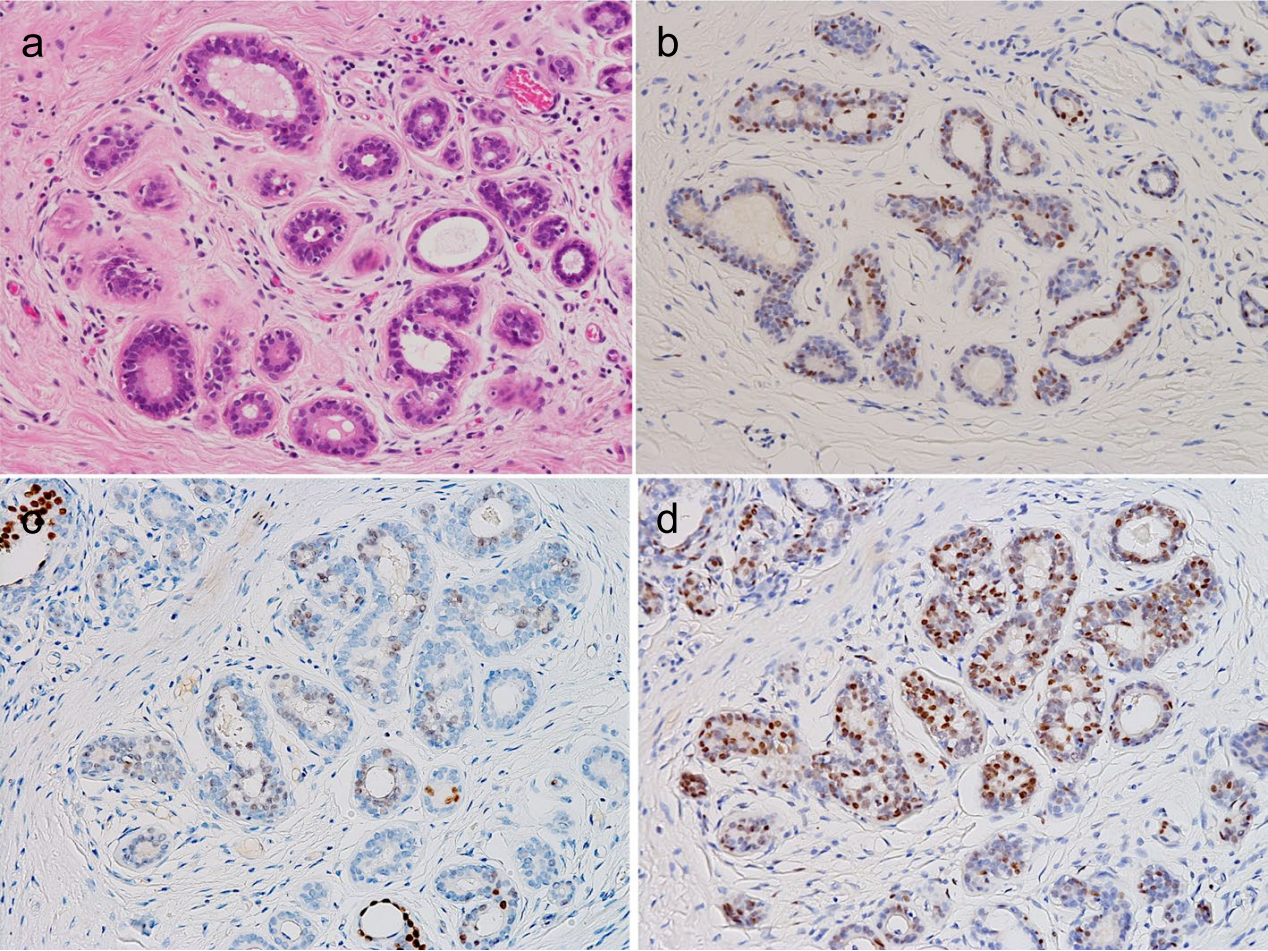
Distribution of SOX9-positive cells in the human non-neoplastic breast. SOX9-positive cells are clearly seen in ducts/acini without particular distribution patterns (**a**, **b**). Seemingly focal co-expression of POU2F3 (**c**) and SOX9 (**d**) is observed (**a**: H&E staining, **b**–**d**: immunohistochemistry)

The above two analyses suggested the relevance of SOX9 for possible “tuft cellness” in the breast. Of note, there has been accumulating evidence of the relevance of SOX9 for breast cancer biology [23–25] and its role as a “master regulator” of TNBCs [26]. Because there have been few studies on SOX9-expressing cells in the human non-neoplastic breast [23], we performed IHC and found that many SOX9-positive cells were present among ductal, acinar, and myoepithelial cells without distinct spatial distribution (Fig. 7a,b). Nevertheless, some ductal/acinar cells appeared to co-express POU2F3 and SOX9 (Fig. 7c,d). After obtaining these data, we performed SOX9-IHC for a recently encountered POU2F3-positive cancer (Case 5 in Table 1) and observed diffuse and strong SOX9 expression (Figure S1).

## Discussion

This study demonstrated that POU2F3 expression occurs in the non-invasive stage of ductal breast carcinomas and rarely in HER2 + /HR + invasive breast carcinomas (so-called triple-positive), while it is prevalent in TNBCs. In the normal breast, our novel findings were the basal and luminal distribution of supposedly two types of POU2F3-positive epithelial cells that possibly contained bona fide tuft cells due to their co-expression of other tuft cell markers. The fact that the previously validated POU2F3-positive/KIT-high/BCL2-high tuft cell-like breast cancers were accompanied by POU2F3-positive DCIS components is strongly reminiscent of HER2 + /HR + , as well as the luminal-type ER + , breast cancers in which invasive and in situ components also share the respective IHC hallmarks [27]. These findings imply that POU2F3 expression can occur as an early event during carcinogenesis in POU2F3-positive breast cancers and, in turn, suggest that POU2F3 expression is possibly related to a distinct type of differentiation (histogenesis) or a distinct cell of origin. Obvious candidate precursors of POU2F3-positive breast cancers could be the (eventually committed) precursors of the rare POU2F3-positive cells in the normal breast. This hypothesis would explain the paucity of POU2F3-positive breast cancers compared with the more frequent luminal-type and HER2-positive/HR-negative breast cancers that are derived from the much more abundant POU2F3-negative epithelial cells.

In light of this hypothesis, in-depth characterization of physiological POU2F3-expressing mammary cells and their precursors appears as a research priority to gain insight into the carcinogenesis of POU2F3-positive breast cancers. In this context, our analyses of normal breast cells/tissues detected small subsets of POU2F3-expressing cells (67 of 390 POU2F3-expressing cells, or 67 of 11,831 epithelial cells) that also co-expressed the other two tuft cell genes, i.e., *SOX9/AVIL* or *SOX9/GFI1B*, at the mRNA level, and a small number of epithelial cells seemed to co-express POU2F3 and SOX9, as shown by IHC. These cells could contain bona fide tuft cells in the breast. However, we also noticed heterogeneity of POU2F3-positive cells at both the mRNA and protein levels. Furthermore, we could not detect cells expressing other representative tuft cell markers, TRPM5 and CHAT, as well as IL-25, the most characteristic cytokine released by tuft cells [22] (data not shown). These results may imply that tuft cells in the breast have substantially different functions from those in other organs or even within the breast, as suggested by tuft cells in the intestines and the airways [22, 28]. In addition, because POU2F3 is expressed in keratinocytes (i.e., a different type of epithelial cells) [29], POU2F3-expressing cells in the breast may contain cells unrelated to tuft cell properties. Further studies are necessary, and the use of upcoming high-resolution spatial transcriptomics or massive high-speed enrichment of POU2F3-positive cells [30] might be helpful to overcome the sensitivity limits of historic scRNA-seq procedures [19].

*SOX9* is a transcription factor generally known for its essential role in cartilage and testis development [31, 32] and also as a tuft cell-related gene [4, 22]. In addition, accumulating studies have indicated a critical role for SOX9 in breast cancer development, maintenance, and progression, particularly in TNBCs [23–26, 33]. To the best of our knowledge, only one previous study by Chakravarty et al. had examined SOX9 protein expression in the non-neoplastic human breast using IHC [23]. Thus, we performed SOX9-IHC on our own samples and found that SOX9-positive cells were widely distributed among ductal, acinar, and myoepithelial cells without any unique distribution pattern (although a partial overlap with POU2F3-positive cells was observed).

We assume that SOX9 expression is not specifically regulated in limited cell types in the breast and that POU2F3 might be a factor involved in SOX9 expression in some epithelial cells, possibly in tuft cells. Although data are lacking to support a direct interaction between POU2F3 and SOX9 in normal tuft cells, a recent SCLC (i.e., cancer) study reported the direct binding of POU2F3 (and its cofactor, POU2AF2) to the *SOX9* gene locus in tuft cell-like SCLC cell lines [34]. In addition, it has been reported that SOX9 maintains the luminal stem/progenitor lineage, to which POU2F3-expressing cells often belonged in our analysis, in normal mammary glands, and SOX9 upregulation could drive luminal-to-basal reprogramming and induce, generally triple-negative, basal-like breast cancers [21, 33]. Consequently, the triple-negative phenotype may be predominant in POU2F3-positive breast cancers. Indeed, one of our POU2F3-positive breast cancers (the only case for which SOX9-IHC was available) clearly expressed SOX9. Larger studies are warranted to validate the reproducibility of our IHC results, in addition to functional studies to address the mechanistic relationship between POU2F3 and SOX9 in the breast. Our SOX9-IHC results seem to contradict the findings by Chakravarty et al., who reported that SOX9 was not expressed in normal ducts and was overexpressed in the cytoplasm in invasive cancers. The reason for the difference is not apparent at this time, but may be related to technical issues, such as antibodies and IHC protocols. Nevertheless, we appreciate this pioneering study reporting that higher SOX9 mRNA expression in breast cancer was associated with worse prognosis [23].

The frequency of POU2F3-positive breast cancers was lower than expected in our new invasive breast carcinoma TMA cohort (0.22%). This finding is consistent with a study by Zhong et al., who recently examined the expression of INSM1, ASCL1, and POU2F3 in 97 breast carcinomas, mainly with neuroendocrine morphology, and reported that only one case was POU2F3 positive [35]. In addition, due to the small number of cases (*n* = 5), the clinical relevance of POU2F3 positivity in breast cancers could not be assessed. Given these limitations, future studies should delineate the clinical, pathological, and molecular features of POU2F3-positive or tuft cell-like breast cancers. To this end, using a recently marketed anti-POU2F3 antibody, unavailable when we performed IHC analyses for cancer TMA tissues, might be helpful. Benign breast tumors could also be included in such studies, as the five fibroadenomas here consistently contained a few POU2F3-positive cells.

## Conclusions

We believe tuft cell-like properties, particularly those related to POU2F3 and SOX9, in neoplastic and non-neoplastic breast tissues are worth further investigation and may be key to dissecting the complex biology of breast cancers, particularly TNBCs. We hope this exploratory study will lay the groundwork for future research.

## Supplementary Information

**Additional file 1: Figure S1.** Pathological features of POU2F3-positive invasive breast carcinomas found in our daily practice. (a–d) Tumor no. 5 (Table 1). This tumor displays the triple-negative subtype (a). The neoplastic cells are positive for POU2F3 (b), BCL2 (c), and KIT (d), and strongly express SOX9 (e) (a: H&E staining; b-e: immunohistochemistry).

**Additional file 2:** **Table S1.** The pathological data of the TMAs. **Table S2.** Expression profiles of POU2F3-expressing cells. **Table S3.** Genes significantly expressed in cells within Clusters 1-2.

## Data Availability

In addition to our own samples, we utilized a publicly available scRNA-seq dataset of normal human breast tissues (GSE164898). The datasets used and/or analyzed during the current study are available from the corresponding author upon reasonable request.

## Abbreviations

DCIS: Ductal carcinoma in situ
ER: Estrogen receptor
H&E: Hematoxylin and eosin
HR: Hormone receptor
IHC: Immunohistochemistry
NSCLC: Non-small cell lung cancer
PR: Progesterone receptor
SCLC: Small cell lung cancer
TMA: Tissue microarray
TNBC: Triple-negative breast cancer
