# Supplementary material for: An exploratory study for tuft cells in the breast and their relevance in triple-negative breast cancer: the possible relationship of SOX9
Source: BMC Cancer. 2023 May 13;23:438. doi: 10.1186/s12885-023-10949-5 (PMC10183142; doi:10.1186/s12885-023-10949-5)

Supplementary information for

An exploratory study for tuft cells in the breast and their relevance in triple-negative breast cancer: the possible relationship of SOX9

Yosuke Yamada, Ronald Simon, Kosuke Iwane, Yuki Nakanishi, Yasuhide Takeuchi, Akihiko Yoshizawa, Masahiro Takada, Masakazu Toi, Hironori Haga, Alexander Marx, Guido Sauter

Supplementary figure legends

Figure S1. Pathological features of POU2F3-positive invasive breast carcinomas found in our daily practice.

(a–d) Tumor no. 5 (Table 1). This tumor displays the triple-negative subtype (a). The neoplastic cells are positive for POU2F3 (b), BCL2 (c), and KIT (d), and strongly express SOX9 (e) (a: H&E staining; b-e: immunohistochemistry).

Figure S1

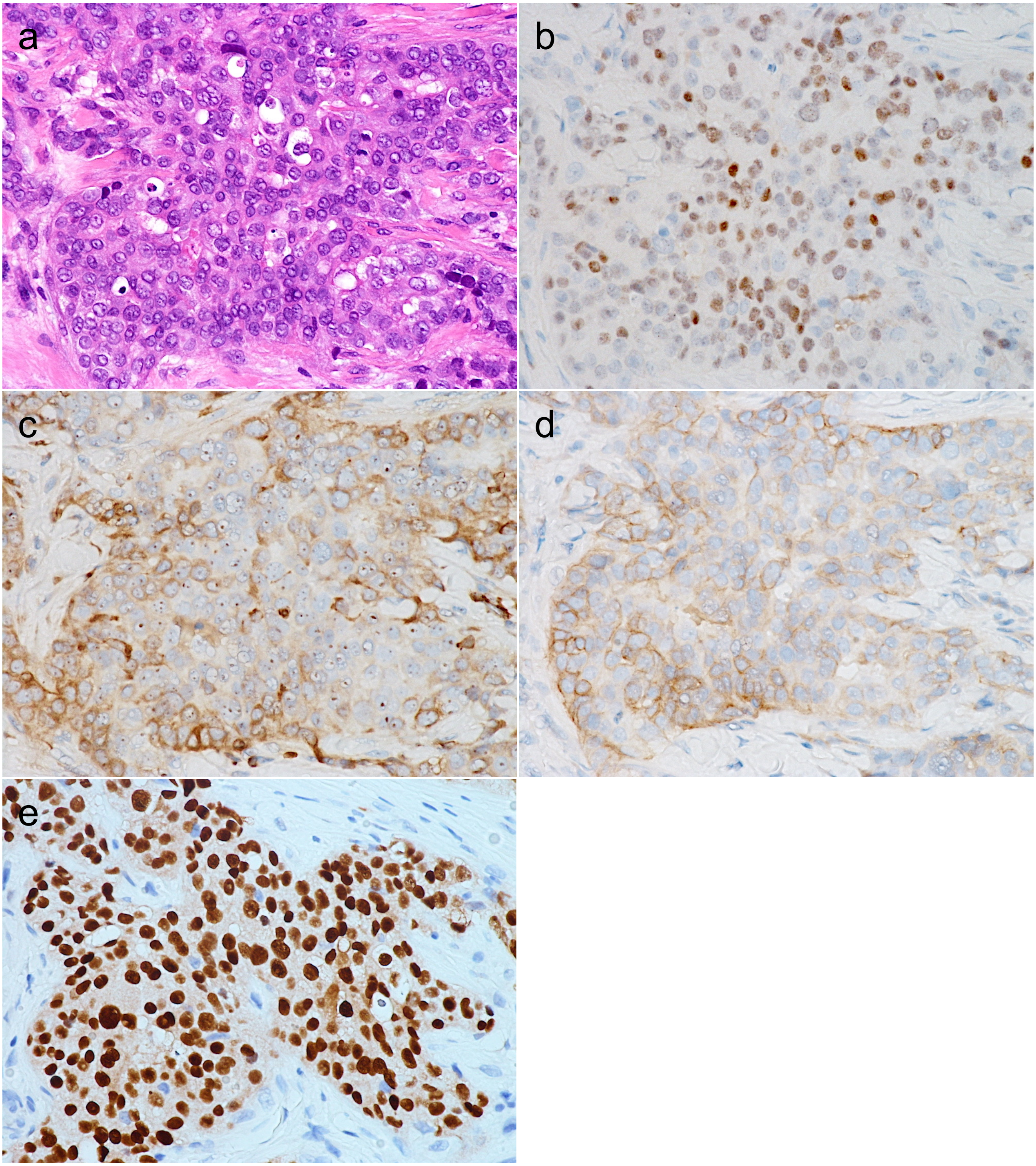

Supplement: Supplementary file 1 — Additional file 1: Figure S1. Pathological features of POU2F3-positive invasive breast carcinomas found in our daily practice. (a–d) Tumor no. 5 (Table 1). This tumor displays the triple-negative subtype (a). The neoplastic cells are positive for POU2F3 (b), BCL2 (c), and KIT (d), and strongly express SOX9 (e) (a: H&E staining; b-e: immunohistochemistry). [file 12885_2023_10949_MOESM1_ESM.pdf]
