# Supplementary material for: An exploratory study for tuft cells in the breast and their relevance in triple-negative breast cancer: the possible relationship of SOX9
Source: BMC Cancer. 2023 May 13;23:438. doi: 10.1186/s12885-023-10949-5 (PMC10183142; doi:10.1186/s12885-023-10949-5)
Supplement: Supplementary file 2 — Additional file 2: Table S1. The pathological data of the TMAs. Table S2. Expression profiles of POU2F3-expressing cells. Table S3. Genes significantly expressed in cells within Clusters 1-2. [file 12885_2023_10949_MOESM2_ESM.zip › table s1.pdf]

Supplementary table legends

Table S1. Pathological features of the breast cancer TMAs

Table S2. Expression profiles of POU2F3-expressing cells

Every POU2F3-expressing cell is numbered as its Cell ID. Clusters indicate the cluster number to which the cell belongs. Expression values are presented after normalization.

Table S3. Genes significantly expressed in cells within clusters 1 and 2

Genes expressed in cells within clusters 1 and 2 were significantly more frequently expressed than cells in other clusters searched. Log_2(clusters 1 and 2/others) indicates the log_2 ratio of (average expression values in cells in clusters 1 and 2)/(those in cells in other clusters). Among the extracted genes, the genes with a log_2 ratio > 0.25 are shown (n = 275). *P*-values and adjusted *P*-values were determined using the Wilcoxon rank sum test and Bonferroni correction, respectively. Adjusted *P*-values < 0.05 were considered to be statistically significant. SOX9 (a tuft cell marker [1]), KIT (a gene frequently expressed in tuft cell-like carcinomas [2]), and CK5 (a basal marker [3]) are included.

References

1. Huang YH, Klingbeil O, He XY, Wu XS, Arun G, Lu B, Somerville TDD, Milazzo JP, Wilkinson JE, Demerdash OE *et al*: **POU2F3 is a master regulator of a tuft cell-like variant of small cell lung cancer**. *Genes Dev* 2018, **32**(13-14):915-928.
2. Yamada Y, Bohnenberger H, Kriegsmann M, Kriegsmann K, Sinn H-P, Goto N, Nakanishi Y, Seno H, Chigusa Y, Fujimoto M *et al*: **Tuft cell-like carcinomas: Novel cancer subsets present in multiple organs sharing a unique gene expression signature**. *British Journal of Cancer* 2022.
3. Badve S, Dabbs DJ, Schnitt SJ, Baehner FL, Decker T, Eusebi V, Fox SB, Ichihara S, Jacquemier J, Lakhani SR *et al*: **Basal-like and triple-negative breast cancers: a critical review with an emphasis on the implications for pathologists and oncologists**. *Mod Pathol* 2011, **24**(2):157-167.
